# Case Report: Perioperative management of ovarian cancer surgery in suspected trousseau's syndrome with recent ischemic stroke and acute myocardial injury

**DOI:** 10.3389/fmed.2026.1882939

**Published:** 2026-09-14

**Authors:** Jiahui Si, Yuxiu Zhang, Jiejin Yang, Dongxin Wang, Ting Ding, Qiongfang Wu

**Affiliations:** 1 Department of Anesthesiology, Peking University First Hospital, Beijing, China; 2 Department of Medical Imaging, Peking University First Hospital, Beijing, China

**Keywords:** cerebral infarction, myocardial injury, ovarian cancer, perioperative management, trousseau's syndrome

## Abstract

**Purpose:**

To describe the perioperative management for ovarian cancer surgery in a patient with suspected Trousseau's syndrome complicated by recent ischemic stroke and acute myocardial injury.

**Case presentation:**

A 43-year-old woman with ovarian cancer developed recurrent cerebral infarctions and acute myocardial injury with dynamic ECG changes during anticoagulation. After multidisciplinary assessment of the competing risks of further surgical delay and intervention, definitive tumor surgery was undertaken under general anesthesia. Postoperatively, delayed emergence and marked troponin elevation occurred without clinically evident new neurological deficit or cardiac, symptomatic cardiac dysfunction, or ongoing cardiovascular instability.

**Conclusion:**

This case illustrates individualized perioperative management in a patient with malignancy-associated hypercoagulability, emphasizing multidisciplinary decision-making, diagnostic restraint, and tailored perioperative care.

## Introduction

Trousseau’s syndrome (TS) is a severe manifestation of cancer-associated thrombosis and is associated with malignancies including pancreatic, gastric, lung, and ovarian cancers (1). It may present with recurrent venous or arterial thromboembolic events and carries a poor prognosis (2). Venous thromboembolism is more common (3), whereas arterial events are less frequently reported (4), with cerebral infarction being a recognized manifestation (2). Concurrent acute ischemic stroke and acute myocardial injury are uncommon and present substantial perioperative challenges.

Current clinical guidelines generally recommend postponing non-emergent surgery after acute ischemic stroke or myocardial infarction because of the increased risk of perioperative risk (5, 6). However, in selected patients with TS, ongoing tumor-associated thrombosis may make further delay unfavorable. We therefore present this case to illustrate individualized perioperative decision-making, emphasizing diagnostic restraint, multidisciplinary risk assessment, and tailored intraoperative management.

## Case presentation

The patient provided written informed consent for publication. She was a 43-year-old Chinese woman (159 cm, 49 kg; unemployed) with no known allergies or regular medications, and a 3-year history of a progressively enlarging left adnexal mass. One month before admission, pelvic ultrasonography showed a 12.5 × 8.2 × 7.3 cm cystic-solid lesion, with elevated CA-125 (333 U/mL) and CA19-9 (1,322 U/mL); Positron emission tomography–computed tomography (PET-CT) suggested ovarian malignancy. She subsequently developed transient aphasia and left upper-limb numbness. Brain magnetic resonance imaging (MRI) revealed acute multifocal cerebral infarctions involving both cerebral hemispheres and the right cerebellum, while magnetic resonance angiography (MRA) showed no large-vessel occlusion. Cervical vascular ultrasonography showed no significant carotid or vertebral stenosis. Contrast-enhanced transthoracic echocardiography (TTE) showed no patent foramen ovale. Autoimmune, vasculitic, and common inherited thrombophilia screening was unremarkable. Anticardiolipin and anti-*β*2-glycoprotein I antibodies were negative, whereas lupus anticoagulant testing on postoperative day (POD) 3 during enoxaparin treatment had limited interpretability. No atrial fibrillation was documented during 24-hour Holter monitoring and lower-extremity venous ultrasonography showed no deep vein thrombosis. D-dimer was elevated at 2.067 mg/L (reference range, 0-0.5 mg/L) without other laboratory findings suggestive of overt disseminated intravascular coagulation (DIC). High-sensitivity cardiac troponin I (hs-cTnI) was within the normal assay-specific reference range, and TTE and electrocardiogram (ECG) (Supplementary Figure 1A) were unremarkable. Enoxaparin 40 mg subcutaneously every 12 h (approximately 0.82 mg/kg) was initiated; the rationale for this dose was not documented. No missed doses were reported and anti-Xa monitoring was not performed.

One month later, she was transferred to our hospital for surgery. Despite ongoing anticoagulation, recurrent cerebral infarctions occurred, with new-onset left facial numbness one day before surgery. Emergency brain MRI revealed multiple new infarcts involving the bilateral frontoparietal and occipital cortices, subcortical white matter, centrum semiovale, and cerebellar hemispheres (Figure 1), while head CT showed no intracranial hemorrhage. The initial aphasia and left upper-limb numbness had improved. Enoxaparin 40 mg every 12 h was continued and withheld the night before surgery. Given the new neurological and cardiac abnormalities occurring shortly before surgery, the dose was not escalated because of perioperative bleeding concerns; the last dose was 18 h before incision. D-dimer decreased from 1.7 mg/L four days before surgery to 0.71 mg/L one day before surgery. Preoperative ECG showed new dynamic ST–T abnormalities predominantly in leads V2–V5 (Supplementary Figure 1B vs. Supplementary Figure 1A), without chest pain or hemodynamic instability. Cardiology considered these changes suggestive of myocardial ischemia, although stroke-related neurogenic changes could not be excluded. hs-cTnI was 108 ng/L (reference 0–11.6 ng/L), while TTE showed preserved left ventricular systolic function without regional wall-motion or valvular abnormalities, and B-type natriuretic peptide remained normal. Coronary evaluation was deferred because it was unlikely to alter immediate management and might delay surgery or complicate antithrombotic management. Transesophageal echocardiography (TEE), dedicated aortic arch imaging and blood culture were not performed.

**Figure 1:**
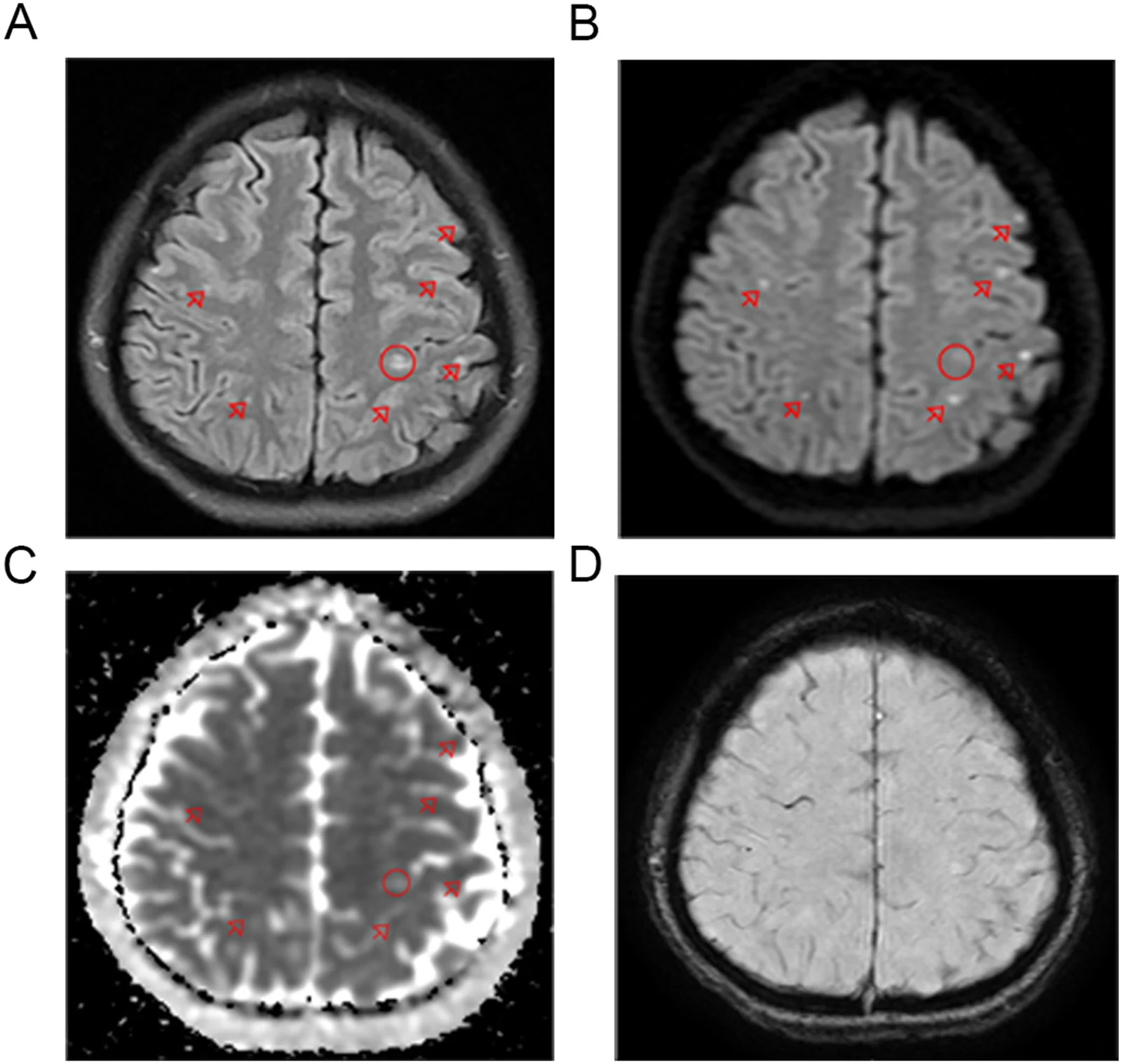
Preoperative brain MRI obtained one day before surgery. **(A–D)** T2-FLAIR, DWI, ADC, and SWI images, respectively. Multiple recent infarcts are seen in the frontal subcortical white matter, appearing hyperintense on **(A)** T2-FLAIR and **(B)** DWI, with corresponding intermediate-to-low signal intensity on **(C)** ADC (arrows). In addition, a chronic ischemic lesion in the left frontal subcortical white matter appears hyperintense on **(A)** T2-FLAIR, isointense on **(B)** DWI, and shows **(C)** no ADC signal reduction (circle). **(D)** SWI shows no abnormal hypointense signal within the brain parenchyma, indicating no susceptibility evidence of intracranial hemorrhage on this examination. MRI, magnetic resonance imaging; T2-FLAIR, T2-weighted fluid-attenuated inversion recovery; DWI, diffusion-weighted imaging; ADC, apparent diffusion coefficient; SWI, susceptibility-weighted imaging.

Given recurrent thromboembolic events during enoxaparin treatment, the multidisciplinary team, including specialists from gynecologic oncology, anesthesiology, neurology, cardiology, hematology, and intensive care, considered malignancy-associated hypercoagulability a major contributor, while alternative embolic mechanisms remained possible. Differential diagnoses included paroxysmal atrial fibrillation, cardiac embolic sources such as nonbacterial thrombotic endocarditis (NBTE), aortic arch disease, antiphospholipid syndrome, inherited thrombophilia, and other autoimmune or hematologic disorders.

Baseline neurological assessment showed an NIH Stroke Scale (NIHSS) score of 1 for left-sided facial numbness, and a modified Rankin Scale (mRS) score of 1. Blood pressure was 119/61 mmHg and heart rate 78 beats/min in sinus rhythm. Routine hematologic, metabolic, coagulation, inflammatory, and renal parameters were within acceptable ranges. The patient was classified as American Society of Anesthesiologists (ASA) physical status IV.

After multidisciplinary evaluation and detailed risk-benefit discussion with the patient and her family, time-sensitive ovarian cancer surgery was scheduled. The decision reflected ongoing thrombotic progression despite anticoagulation, the potentially reversible hypercoagulable state after tumor treatment, relatively stable neurological and cardiovascular status, and concern that further delay might increase risk. Continued anticoagulation, further diagnostic evaluation, neoadjuvant chemotherapy, or biopsy alone were considered but not selected because they were not expected to address the immediate concern as promptly as definitive tumor treatment.

### Intraoperative management

Upon arrival in the operating room, the patient received oxygen via face mask at 5 L/min. Monitoring included invasive arterial blood pressure, pulse oximetry, ECG with ST-segment surveillance, end-tidal carbon dioxide, core temperature, urine output, train-of-four neuromuscular monitoring, cardiac index (CI) and stroke volume variation (SVV) using the FloTrac/Vigileo system (Edwards Lifesciences), processed electroencephalography (SedLine®) for anesthetic depth, and near-infrared spectroscopy (NIRS; Masimo O3™) for regional cerebral oxygen saturation (rSO₂). Baseline right and left frontal rSO₂ values were 68% and 67%.

General anesthesia was induced with ciprofol 25 mg, sufentanil 25 μg, and rocuronium 40 mg after initiation of remifentanil target-controlled infusion (effect-site concentration 2 ng/mL), followed by uneventful tracheal intubation. Anesthesia was maintained with sevoflurane 1%, propofol 4–6 mg/kg/h, and remifentanil 2–4 ng/mL. Dexmedetomidine 40 μg was infused over 30 min after induction. Ciprofol was used for induction for hemodynamic stability, while propofol was used for maintenance because of its rapid titratability. An ultrasound-guided transversus abdominis plane block was performed with 40 mL of 0.375% ropivacaine.

Predefined individualized intraoperative goals included maintaining systolic blood pressure (SBP) close to baseline as the primary goal, preferably within ±10% when feasible, and mean arterial pressure (MAP) > 65mmHg as a minimum safety threshold; avoiding sustained tachycardia; maintaining normocapnia, normothermia, and acid–base and electrolyte balance; and correcting anemia, hypovolemia, or excessive anesthetic depth if perfusion concerns arose. Goal-directed fluid management was guided by CI and SVV, with SVV maintained <13% when clinically interpretable. An absolute rSO₂ < 50% or >20% decline from baseline was used as the intervention threshold. Norepinephrine (0.01–0.04 μg/kg/min; total 600 μg) was titrated to limit hypotension, and isosorbide mononitrate (0.5–1.0 μg/kg/min; total 13.9 mg) to reduce myocardial oxygen demand and support coronary perfusion. Both were administered from induction until departure from the operating room over approximately 5 h 20 min. New ST-segment changes, arrhythmia, sustained hypotension, or clinically relevant deterioration in CI or SVV prompted reassessment, with TEE and cardiology consultation considered if abnormalities persisted.

The procedure comprised open total hysterectomy with bilateral salpingo-oophorectomy, pelvic and para-aortic lymphadenectomy, omentectomy, appendectomy, lysis of intestinal adhesions, ureterolysis, gonadal veins ligation, and multiple peritoneal biopsies, lasting 3 h 41 min. Intraoperative SBP ranged from 115 to 160 mmHg, MAP from 60 to 80 mmHg, and heart rate from 60 to 80 beats/min. CI ranged from 3.0 to 4.4 L/min/m^2^, and SVV ranged from 3% to 9%. MAP <65 mmHg lasted a cumulative 3 min. SBP exceeded 110% of baseline for approximately 11 min, mainly during skin incision, intra-abdominal manipulation, and tracheal extubation, and was managed by adjusting anesthetic depth and vasoactive therapy. The lowest right and left rSO₂ values were both 62%, without reaching the intervention threshold. The processed EEG index ranged from 25 to 46 (reference range 25–50). No worsening ST-segment changes or clinically significant arrhythmias occurred. The lowest hemoglobin was 11.1 g/dL, peak lactate 2.56 mmol/L, and core temperature 36.1–36.5 °C. During the peri-anesthetic period, 4,500 mL crystalloid and 500 mL colloid were administered under goal-directed hemodynamic management; estimated blood loss was 500 mL, urine output 600 mL, and no transfusion was required.

After discontinuation of anesthetic agents and administration of sugammadex 200 mg, tracheal extubation was delayed by approximately 30 min because of delayed emergence. No residual neuromuscular blockade, metabolic or respiratory abnormality, hypothermia, or new neurological deterioration was identified. After extubation, the patient remained drowsy but arousable with stable vital signs and was transferred to the intensive care unit, regaining full consciousness within 3 h.

### Postoperative course

The postoperative antithrombotic regimen was adjusted according to the evolving balance between thrombosis and bleeding. Enoxaparin 40 mg every 12 h was resumed 6 h after surgery once hemostasis was confirmed because of the high risk of recurrent thrombosis, and increased to 60 mg every 12 h on POD 2 because of persistent thrombotic risk in the absence of clinically significant bleeding. Postoperative body weight was unavailable, and anti-Xa monitoring was not performed. Aspirin was subsequently added for the arterial thrombotic phenotype. After 100 mL of vaginal bleeding on POD 14, anticoagulation was temporarily withheld and changed on POD 15 to enoxaparin 30 mg every 12 h plus clopidogrel 75 mg daily. The bleeding source and relevant examination findings were unavailable, and a causal relationship with intensified anticoagulation could not be established. At discharge, rivaroxaban 20 mg once daily was prescribed for at least 6 months for long-term anticoagulation in the setting of recurrent arterial thromboembolic events and suspected malignancy-associated hypercoagulability, rather than specifically for NBTE. An oral regimen was selected to facilitate long-term outpatient treatment and adherence. Clopidogrel 75 mg once daily was prescribed by cardiology for at least 3 months as single-antiplatelet therapy for secondary prevention of arterial thrombotic events.

Postoperative drain output was 280 mL on POD 1 and decreased to < 20 mL/day thereafter. Hemoglobin reached a nadir of 80 g/L on POD 7 and increased to 117 g/L before discharge; no blood products were administered. Serial postoperative ECGs showed evolving ST–T abnormalities with prominent T-wave inversion in the anterior precordial leads on POD 1 (Supplementary Figure 1C), followed by gradual resolution toward baseline by POD 12 (Supplementary Figure 1D vs. Supplementary Figure 1A). hs-cTnI peaked at 5,226.3 ng/L on POD 1 and declined to 21 ng/L by POD 12, while TTE on POD 13 remained unremarkable. Although these changes raised concern for myocardial ischemia, the ECG findings were not sufficiently specific to distinguish coronary ischemic from stroke-related neurogenic myocardial injury. Further coronary and cardiovascular evaluation was deferred because the patient remained clinically stable and had postoperative bleeding risk. The event was therefore described as postoperative acute myocardial injury in the context of pre-existing myocardial injury. Its mechanism remained uncertain, with possible contributions from progression of the pre-existing injury, an ischemic mechanism, or stroke-related neurogenic myocardial injury.

The patient was discharged on POD 19 without new neurological deficit, symptomatic cardiac dysfunction, or ongoing cardiovascular instability (NIHSS 1 for residual left facial numbness; mRS 1). Histopathology confirmed ovarian endometrioid carcinoma with a clear cell component (FIGO stage IA). Postoperative D-dimer levels showed an overall downward trend. Adjuvant chemotherapy with paclitaxel 240 mg plus carboplatin 550 mg every 21 days was initiated on POD 18.

At the four-month telephone follow-up, no recurrent neurological, cardiovascular, or clinically apparent thromboembolic events were reported. Left facial numbness had completely resolved, the patient remained independent in daily activities (estimated mRS 0), with no further major bleeding or clinical evidence of oncologic progression. She was taking rivaroxaban alone. Postoperative brain imaging was not performed; therefore, silent cerebral infarction or asymptomatic radiological progression could not be assessed.

A timeline of the key clinical events is provided in Table 1, with detailed perioperative findings in Supplementary Table 1.

**Table 1:** Timeline of key clinical events.

Time relative to surgery	Key clinical events
3 years before surgery	Progressive enlargement of a left adnexal mass.
∼1 month before surgery	Adnexal mass with elevated CA-125/CA19-9; PET-CT suggested ovarian malignancy. Transient aphasia and left arm numbness; MRI showed multifocal cerebral infarctions. Enoxaparin initiated.
1 day before surgery	D-dimer decreased from 1.7 to 0.71 mg/L. New left facial numbness; recurrent multifocal infarctions on MRI; new ST–T changes in V2–V5; hs-cTnI 108 ng/L.
Preoperative assessment	Multidisciplinary evaluation supported time-sensitive tumor surgery after risk–benefit discussion with the patient and family.
Day of surgery	Enoxaparin withheld for 18 h. Definitive ovarian cancer surgery performed under general anesthesia with individualized hemodynamic and cerebral oxygenation management.
POD 1–3	Enoxaparin resumed and intensified. hs-cTnI peaked at 5,226.3 ng/L with evolving ST–T abnormalities; hemoglobin decreased to 87 g/L.
POD 12–15	hs-cTnI declined to 21 ng/L and ECG abnormalities improved. Vaginal bleeding prompted temporary interruption and modification of antithrombotic therapy.
POD 18–19	Pathology confirmed FIGO IA ovarian endometrioid carcinoma with clear cell component. Adjuvant chemotherapy initiated. Discharged on POD 19 without new neurological deficit, symptomatic cardiac dysfunction, or ongoing cardiovascular instability.
4-month follow-up	No recurrent clinically apparent thromboembolic, neurological, or cardiovascular events; independent functional status and no reported oncologic progression.

ECG, electrocardiogram; hs-cTnI, high-sensitivity cardiac troponin I; MRI, magnetic resonance imaging; PET-CT, positron emission tomography–computed tomography; POD, postoperative day.

### Patient perspective

After multidisciplinary discussion of the risks of surgery and further delay, the patient and her family understood the potential complications and elected to proceed with surgery. At follow-up, the patient reported a positive experience with the multidisciplinary decision-making process and perioperative care.

## Discussion

Trousseau's syndrome is a severe manifestation of malignancy-associated hypercoagulability associated with substantial early mortality (1). Its reported prevalence ranges from 1% to 11% in specific populations, and thromboembolic events may precede cancer diagnosis (7). In lung cancer, up to 52.6% of patients with TS have been reported to die within 90 days (2). The pathogenesis is multifactorial, involving Virchow's triad, tumor-derived procoagulant factors, platelet activation, hypoxia, and systemic inflammation. Diagnosis relies on the clinical presentation and imaging findings with exclusion of alternative causes; elevated D-dimer is supportive but nonspecific (1). Low-molecular-weight heparin remains an important anticoagulant, whereas direct oral anticoagulants require individualized assessment (8). Perioperative reports involving active malignancy with recent ischemic stroke and acute myocardial injury remain limited and heterogeneous (9–12). This case illustrates the challenge of balancing recurrent malignancy-associated thrombosis against the perioperative risks of definitive cancer surgery.

Diagnostic restraint was essential when interpreting the thromboembolic events. The combination of ovarian malignancy, recurrent multifocal cerebral infarctions, elevated D-dimer, and thrombotic progression during anticoagulation supported suspected TS and a malignancy-associated thromboembolic mechanism. However, NBTE and aortic embolic sources remained plausible but could not be confirmed or excluded because TEE, dedicated aortic imaging, and blood cultures were not performed. No atrial fibrillation was documented on 24-hour Holter monitoring, although intermittent atrial fibrillation remained possible. Autoimmune, vasculitic, and common inherited thrombophilia screening was unremarkable; anticardiolipin and anti-*β*2-glycoprotein I antibodies were negative, while lupus anticoagulant testing was limited by concurrent enoxaparin use. Thus, alternative embolic and hypercoagulable mechanisms remained unresolved.

According to the *Fifth Universal Definition of Myocardial Infarction* (13), the dynamic troponin elevation met criteria for acute myocardial injury, whereas myocardial infarction requires additional evidence of acute ischemia. Although cardiology considered the dynamic ST–T abnormalities concerning for myocardial ischemia, they were not sufficiently specific to distinguish an ischemic mechanism from stroke-related neurogenic myocardial injury. An ischemic contribution to the postoperative troponin rise could not be excluded; however, the event was not classified as myocardial injury after noncardiac surgery (MINS) because myocardial injury was already present preoperatively and a competing neurogenic mechanism remained plausible. In the absence of ischemic symptoms, coronary imaging, or cardiac MRI, and with unremarkable echocardiographic findings, myocardial infarction could not be definitively established. In the setting of active ovarian cancer and suspected TS, malignancy-associated coronary microvascular thrombosis was considered possible, but coronary embolism, oxygen supply–demand mismatch, plaque-related coronary events, stroke-related neurogenic myocardial injury and other cancer-associated cardiovascular mechanisms remained in the differential.

The principal educational value of this case lies in the decision-making process. Current guidelines generally recommend delaying non-emergent surgery after acute myocardial infarction and avoiding elective surgery within 30 days after ischemic stroke, with risk declining after 90 days (5, 6). Although some evidence suggests that, when surgery is unavoidable within 2 weeks of stroke, procedures performed within the first 3 days may carry lower risk than those performed on days 4–14 (14), the present surgery was undertaken within 1 day of the latest ischemic event despite its high perioperative risk. The decision was based on ongoing thromboembolism despite anticoagulation, a potentially reversible malignancy-associated hypercoagulable state, clinical stability, and multidisciplinary risk assessment of further delay. These considerations were case-specific and should not be interpreted as general criteria for early surgery.

Anesthetic management focused on balancing cerebral and myocardial oxygen supply and demand. NIRS was used as an adjunctive monitor of regional frontal cerebral oxygenation, with rSO₂ decrease to <80% of baseline or an absolute value <50% considered a potential desaturation threshold (15). However, NIRS reflects only regional cortical oxygenation rather than all cerebral territories, and evidence that NIRS-guided management improves outcomes after noncardiac surgery remains limited (16). The INPRESS trial supports individualized blood pressure management near baseline (17), which may be relevant after recent cerebral ischemia because of impaired autoregulation, while excessive hypertension may increase myocardial oxygen demand. Hemodynamic management therefore aimed to balance cerebral perfusion and myocardial oxygen demand rather than target a universal blood pressure threshold. Norepinephrine and isosorbide mononitrate were carefully titrated according to arterial pressure and clinical status. The use of adjuvant dexmedetomidine may exert potential but unproven neuroprotective and cardioprotective effects in this patient (18). Overall, these measures represented an individualized rather than standardized strategy.

Although tumor removal may have reduced the malignancy-associated prothrombotic stimulus, its specific contribution cannot be determined because antithrombotic therapy was also modified. Likewise, the effectiveness of individualized hemodynamic and cerebral oxygenation management cannot be established from a single case.

This report has several limitations. First, the mechanism of recurrent cerebral infarction remained uncertain because blood cultures, TEE, and dedicated aortic imaging were not performed; therefore, NBTE and aortic embolic sources could not be excluded. Lupus anticoagulant testing was performed during enoxaparin treatment, limiting its interpretation. Second, Second, anti-Xa monitoring was not performed, limiting assessment of anticoagulation intensity, and the postoperative antithrombotic regimen was an empirical individualized strategy rather than an evidence-based or NBTE-specific treatment. Third, the mechanism of myocardial injury remained uncertain because coronary imaging and cardiac MRI were unavailable and stroke-related neurogenic myocardial injury could not be excluded. Fourth, NIRS assessed only regional frontal oxygenation, and postoperative brain imaging and standardized neurological and oncologic assessments were lacking. The favorable outcome should therefore not be interpreted as evidence supporting routine surgery after recent cerebral infarction or myocardial injury.

In conclusion, this case does not establish the safety or effectiveness of early surgery after stroke or acute myocardial injury. Rather, it illustrates individualized decision-making in a patient with suspected malignancy-associated hypercoagulability and recurrent thrombosis during anticoagulation. In this patient, tumor resection followed multidisciplinary assessment, with perioperative management focused on cerebral and myocardial oxygen balance and thrombotic–bleeding risk.

## Data Availability

The original contributions presented in the study are included in the article/Supplementary Material, further inquiries can be directed to the corresponding author.

## Supplementary material

SUPPLEMENTARY FIGURE 1 Serial electrocardiographic changes and perioperative hs-cTnI dynamics. (**A**) Baseline ECG obtained one month before surgery, when hs-cTnI was within the normal range. (**B**) ECG obtained one day before surgery showing new ST–T abnormalities predominantly in the V2-V5 leads compared with baseline; hs-cTnI was 108 ng/L. (**C**) ECG obtained on POD1 showing further evolution of the ST–T abnormalities, with prominent T-wave inversion in the anterior precordial leads; hs-cTnI peaked at 5,226.3 ng/L on the same day. (**D**) ECG obtained on POD 12 showing marked improvement of the ST–T abnormalities toward baseline, with hs-cTnI declining to 21 ng/L. All ECGs were recorded at 25 mm/s and 10 mm/mV. ECG, Electrocardiography; POD, postoperative day; hs-cTnI, high-sensitivity cardiac troponin I.
