# Supplementary material for: Case Report: Perioperative management of ovarian cancer surgery in suspected trousseau's syndrome with recent ischemic stroke and acute myocardial injury
Source: Front Med (Lausanne). 2026 Sep 14;13:1882939. doi: 10.3389/fmed.2026.1882939 (PMC13616941; doi:10.3389/fmed.2026.1882939)
Supplement: Supplementary file 1 [file Table1.docx]

**Supplementary Table 1. Detailed perioperative clinical, laboratory, and imaging findings**

| **Time point** | **Findings** |
| --- | --- |
| **Preoperative** | |
| ~1 month before surgery  (local hospital) | **Pelvic ultrasonography:** Cystic-solid left adnexal lesion, 12.5 × 8.2 × 7.3 cm.  **Tumor markers:** CA-125 333 U/mL; CA19-9 1,322 U/mL.  **PET-CT:** Findings suggestive of ovarian malignancy.  **Brain MRI/MRA:** Acute multifocal cerebral infarctions involving both cerebral hemispheres and the right cerebellum; no large-vessel occlusion.  **Cervical vascular ultrasonography**: No significant stenosis or abnormal flow velocity in the bilateral common carotid, internal carotid, external carotid, or vertebral arteries.  **Contrast-enhanced TTE:** No patent foramen ovale.  **Bilateral lower-extremity venous ultrasonography**: No deep vein thrombosis was detected.  **Autoimmune and vasculitic screening:** Negative. Testing included anti-CCP antibodies, ANA profile, and ANCA.  **Antiphospholipid screening:** Anticardiolipin and anti-β2-glycoprotein I antibodies were negative. Lupus anticoagulant testing was performed on POD 3 during enoxaparin treatment and therefore had limited interpretability.  **Thrombophilias screening:** Protein C, protein S, and antithrombin activities were within normal ranges, and no activated protein C resistance was detected.  **24-h Holter monitoring:** Sinus rhythm with premature atrial contractions and short runs of atrial tachycardia; no atrial fibrillation documented.  **Coagulation:** D-dimer 2.067 mg/L (reference 0–0.5 mg/L); platelets 230 × 10⁹/L; fibrinogen 2.07 g/L; PT 11.2 s; aPTT 23.4 s.  **Cardiac assessment:** hs-cTnI 14 ng/L (reference <34 ng/L; Siemens Atellica platform); ECG and TTE unremarkable. |
| 1–2 days before surgery  (our hospital) | **Brain MRI/CT (Pre-op day 1):** Multiple new infarcts involving the bilateral frontoparietal and occipital cortices, subcortical white matter, centrum semiovale, and cerebellar hemispheres; no intracranial hemorrhage on CT.  **Neurologic assessment:** Left facial numbness; NIHSS 1 and mRS 1.  **ECG/hs-cTnI (Pre-op day 1):** New dynamic ST–T abnormalities predominantly in the V2-V5 leads without chest pain or hemodynamic instability; hs-cTnI 108 ng/L (reference 0–11.6 ng/L; microparticle chemiluminescence immunoassay, Beckman Coulter DxI 800; measured 23 h before surgery).  **TTE/BNP:** Preserved left ventricular systolic function without regional wall-motion abnormalities; BNP within the normal range.  **D-dimer:** 1.7 mg/L at 4 days (outpatient) and 0.71 mg/L at 1 day before surgery (reference 0–0.5 mg/L).  **Tumor markers:** CA-125 2,623 U/mL; CA19-9 6,397 U/mL.  **Hematology/coagulation:** Hemoglobin 110 g/L; platelets 235 × 10⁹/L; fibrinogen 1.97 g/L; PT 12.3 s; aPTT 26 s.  **Other laboratory findings:** CRP 3.59 mg/L; creatinine 65.3 μmol/L; eGFR 99.8 mL/min/1.73 m². |
| **Intraoperative** | |
| During surgery | **Baseline:** BP 119/61 mmHg; HR 78 beats/min in sinus rhythm; right/left frontal rSO₂ 68%/67%.  **Arterial blood gas:** Before induction—hemoglobin 117 g/L, glucose 6.6 mmol/L, PaCO₂ 35.1 mmHg, PaO₂ 260.4 mmHg, lactate 1.54 mmol/L; 30 min before the end of surgery—hemoglobin 121 g/L, glucose 7.3 mmol/L, PaCO₂ 41.3 mmHg, PaO₂ 307.3 mmHg, lactate 2.56 mmol/L.  **Hemodynamics:** SBP 115–160 mmHg; MAP 60–80 mmHg; HR 60–80 beats/min; cumulative MAP <65 mmHg, 3 min; cumulative SBP > 110% of baseline, 11 min; CI 3.0–4.4 L/min/m²; SVV 3%–9%.  **Neuromonitoring/cardiac monitoring:** Lowest right/left rSO₂ 62%/62%; processed EEG index 25–46; no worsening ST-segment changes or clinically significant arrhythmias.  **Other intraoperative findings:** Core temperature 36.1–36.5°C; estimated blood loss 500 mL; urine output 600 mL; 4,500 mL crystalloid and 500 mL colloid; no intraoperative transfusion. |
| **Postoperative** | |
| POD 0 | **hs-cTnI:** 653.2 ng/L (20:56).  **Laboratory findings:** Hemoglobin 100 g/L; lactate 1.46 mmol/L; D-dimer 3.42 mg/L. |
| POD 1 | **hs-cTnI:** 2,319.8 ng/L (04:49), peak 5,226.3 ng/L (15:54), and 4,235.2 ng/L (20:18).  **ECG:** Further evolution of dynamic ST–T abnormalities with prominent T-wave inversion in the anterior precordial leads.  **Bedside lower-extremity vascular ultrasonography:** no evidence of deep vein thrombosis in either leg.  **Other findings:** D-dimer 2.37 mg/L; hemoglobin 110 g/L; drain output 280 mL. |
| POD 2 | **hs-cTnI:** 3,376.3 ng/L (00:19), 1,936.8 ng/L (04:28), and 1,706.2 ng/L (14:56).  **ECG:** Persistent dynamic ST–T abnormalities in the anterior precordial leads.  **Other findings:** D-dimer 0.79 mg/L; hemoglobin 106 g/L. |
| POD 3 | **hs-cTnI:** 852.8 ng/L.  **ECG:** Persistent dynamic ST–T abnormalities in the anterior precordial leads.  **Laboratory findings:** Hemoglobin 87 g/L; D-dimer 0.62 mg/L.  **Lupus anticoagulant (SCT):** Negative, but interpretation was limited by concurrent enoxaparin use; the interval from the last dose to sampling and SCT heparin-neutralizer information were unavailable. |
| POD 4–11 | **Hemoglobin:** 91 g/L (POD 4), 80 g/L (POD 7), and 87 g/L (POD 11).  **D-dimer:** 0.73 mg/L on POD 4. |
| POD 12–13 | **POD 12:** hs-cTnI 21 ng/L; ECG abnormalities markedly improved toward baseline.  **POD 13:** TTE showed no significant abnormalities; D-dimer 0.88 mg/L; hemoglobin 94 g/L. |
| POD 14–19 | **Hemoglobin:** 117 g/L (POD 18).  **Clinical course:** Vaginal bleeding of approximately 100 mL occurred on POD 14; the patient was discharged on POD 19 without new neurological deficit, symptomatic cardiac dysfunction, or ongoing cardiovascular instability (NIHSS 1; mRS 1). |
| **Histopathology** | |
| Definitive pathology | **Diagnosis:** Ovarian endometrioid carcinoma with a clear cell component; FIGO stage IA; tumor grade G3; residual disease R0.  **Staging findings:** Lymph-node and peritoneal findings negative; cytology/ascites findings not specified.  **Immunohistochemistry:** p53 wild-type; pMMR (MLH1/MSH2/MSH6/PMS2 intact); ER 70% positive; PgR 70% positive; Ki-67 60%. |

**Abbreviations:** ANA, antinuclear antibody; ANCA, antineutrophil cytoplasmic antibody; anti-CCP, anti-cyclic citrullinated peptide; aPTT, activated partial thromboplastin time; BNP, B-type natriuretic peptide; BP, blood pressure; CRP, C-reactive protein; DBP, diastolic blood pressure; ECG, electrocardiogram; eGFR, estimated glomerular filtration rate; ER, estrogen receptor; hs-cTnI, high-sensitivity cardiac troponin I; HR, heart rate; MAP, mean arterial pressure; MRA, magnetic resonance angiography; MRI, magnetic resonance imaging; mRS, modified Rankin Scale; NIHSS, National Institutes of Health Stroke Scale; PET-CT, positron emission tomography-computed tomography; PgR, progesterone receptor; pMMR, proficient mismatch repair; POD, postoperative day; Pre-op, preoperative; PT, prothrombin time; rSO₂, regional cerebral oxygen saturation; SBP, systolic blood pressure; SCT, silica clotting time; TTE, transthoracic echocardiography; CI, cardiac index; SVV, stroke volume variation.
